# Adiponectin promotes syncytialisation of BeWo cell line and primary trophoblast cells

**DOI:** 10.1186/1477-7827-8-128

**Published:** 2010-10-29

**Authors:** Delphine Benaitreau, Esther Dos Santos, Marie-Christine Leneveu, Philippe De Mazancourt, René Pecquery, Marie-Noëlle Dieudonné

**Affiliations:** 1 Université de Versailles-St Quentin, Service de Biochimie et Biologie Moléculaire, UPRES-EA 2493, Faculté de Médecine Paris-Ile de France Ouest, PRES Universud Paris, Centre Hospitalier de Poissy-Saint Germain, 78303 Poissy Cedex, France

## Abstract

**Background:**

In human pregnancy, a correct placentation depends on trophoblast proliferation, differentiation, migration and invasion. These processes are highly regulated by placental hormones, growth factors and cytokines. Recently, we have shown that adiponectin, an adipokine, has anti-proliferative effects on trophoblastic cells. Here, we complete this study by demonstrating that adiponectin modulates BeWo and human villous cytotrophoblast cell differentiation.

**Results:**

We showed that hCG secretion was up-regulated by adiponectin treatment in both BeWo cells and human cytotrophoblasts from very early placentas (5-6 weeks). The expression of two trophoblast differentiation markers, leptin and syncytin 2, was also up-regulated by adiponectin in BeWo cells. Moreover, adiponectin treatment induced a loss of E-cadherin staining in these cells. In parallel, we demonstrated that AdipoR1 and AdipoR2 are up-regulated during forskolin induced BeWo cell differentiation, reinforcing the role of adiponectin in trophoblast syncytialization. SiRNA mediated down-regulation of AdipoR1 and AdipoR2 was used to demonstrate that adiponectin effects on differentiation were essentially mediated by these receptors. Finally, using a specific inhibitor, we demonstrated that the PKA signalling pathway could be one pathway involved in adiponectin effects on trophoblast differentiation.

**Conclusion:**

Adiponectin enhances the differentiation process of trophoblast cells and could thus be involved in functional syncytiotrophoblast formation.

## Background

In human pregnancy, trophoblast cells play an essential role in embryo implantation and placental development. These cells differentiate according to one of two distinct pathways. In the extravillous pathway, cytotrophoblasts (CT) proliferate, differentiate into an invasive phenotype, and penetrate into the maternal decidua and myometrium [1,2]. In the villous pathway, mononuclear CT fuse to form a specialized multinuclear syncytium called syncytiotrophoblast (ST) on the outer layer of placental villi [1]. ST formation plays an important role in human placentation. This process might be affected in some pathological pregnancy situations. For example, altered ST formation was observed in human preeclampsia [2].

The ST layer is the site of many placental functions necessary for foetal growth and development, including nutrient, gas exchanges, and synthesis of steroid and peptide hormones [2]. Characteristics related to trophoblast differentiation include the production of hormones like human chorionic gonadotropin (hCG), human placental lactogen, and leptin [3]. However, morphological changes, which involve fusion of CT to form the ST layer represent a hallmark of this differentiation. Studies have highlighted the impact of adhesion molecules such as cadherins in trophoblast differentiation. Among these, E-cadherin is localized at the membrane of the isolated CT and disappears when the CT fuse into ST [4,5]. Very recently, studies have demonstrated the role of former envelope viral proteins derived from human endogenous retrovirus (HERVs) in trophoblast cell fusion, of which syncytin-1 [6] and syncytin-2 [7] seem to be of high importance. Moreover, syncytin-2 mRNA and protein are particularly expressed in the ST [7,8].

Different *in vitro *studies have shown that the villous CT differentiation could be modulated by hormones and by soluble factors. For example, epidermal growth factor (EGF) [9], 17β-estradiol [10], granulocyte macrophage-colony stimulating factor (GM-CSF) [11], glucocorticoids [12], and hCG [13] induce differentiation, whereas tumor necrosis factor α (TNFα) [2,14] and tumor growth factor β1 (TGFβ1) [15] impair this process. Adipokines such as leptin and adiponectin have recently been shown to affect the reproductive system through central effects on the hypothalamus and/or peripheral effects on the ovary, endometrium, or directly on the embryo and placenta developments [16-21]. Indeed, leptin is specifically expressed in the ST [18], and is considered as a new placental hormone [18,22]. Adiponectin is a cytokine, predominantly produced by adipose tissue, and present at high concentrations in human circulation (5-15 μg/ml) [23]. This adipokine is described as an insulin sensitizing hormone [24-26], and has been shown to have anti-inflammatory, anti-angiogenic, anti-atherosclerotic and anti-proliferative roles in various cell types [25]. Adiponectin is a 30 kDa protein that is assembled into an array of complexes composed of adiponectin multimers. Adiponectin subunits assemble into trimers called low molecular weight complexes (LMW), hewamers or middle molecular weight forms (MMW), or more elaborate high molecular weight complexes (HMW) composed of 9 hewamers. The HMW form is predominant in human circulation [27]. Two specific adiponectin receptors, AdipoR1 and AdipoR2 have been identified [28]. Both receptors contain seven transmembrane domains but are structurally and functionally distinct from G-protein coupled receptors. AdipoR1 and AdipoR2 are both expressed in human endometrium and placenta [19,29,30]. However, adiponectin is only produced by endometrial cells at the foetal-maternal interface [19]. An additional receptor for adiponectin, T-cadherin, has recently been described [31] but is not expressed in human trophoblast [30]. AdipoR1 and AdipoR2 activate different signal transduction pathways such as the AMPK, PKA, PI3K and P38/P42/P44 MAPK pathways [16,25,28,32]. Recently, we have shown that adiponectin exerts anti-proliferative effects on trophoblastic cell lines (JEG-3 and BeWo) and also on human trophoblasts [30]. Moreover, it has been shown that adiponectin serum concentrations are deregulated in some placental pathologies as gestational diabetes mellitus [33], and preeclampsia [34,35]. However, to date, there are no data concerning the direct impact of adiponectin in trophoblast differentiation.

To study adiponectin effects on trophoblast differentiation, the widely used trophoblast differentiation model BeWo choriocarcinoma cell line was chosen [36,37]. These cells have indeed a high degree of similarity to normal placental trophoblasts and can morphologically and functionally differentiate *in vitro *into ST. In particular, BeWo differentiation can be strongly induced by cAMP analogs or forskolin, an adenylate cyclase activator [37,38]. Thus, the effects of adiponectin on differentiation in both BeWo cells and in villous cytotrophoblasts were tested by measuring hCG secretion and expression of various differentiation markers (leptin, syncytin-2 and E-cadherin) to evaluate the associated morphological and biochemical changes.

## Methods

### Materials

The culture medium DMEM/F12, penicillin, streptomycin, forskolin, Compound C, H89, and bovine serum albumine (BSA) were purchased from Sigma Chemical Co. (St Louis, Mo, USA). Fetal Calf Serum (FCS) was purchased from Gibco (Invitrogen, Carlsbad, Ca, USA). Recombinant human adiponectin was provided by R&D Systems Europe Ltd (Abingdon, UK), Superscript II Rnase H- RT by Invitrogen corporation (Carlsbad, Ca, USA), hCG by Organon (Puteaux, France), and RNAguard by Pharmacia Biotechnology (Uppsala, Sweden). Trypsin was provided by Difco Laboratories (Detroit, Mi, USA). The origin of the different antibodies used are described in the following paragraphs.

### BeWo cell culture

The human choriocarcinoma cell line BeWo was obtained from American Type Collection of Cell (Manassas, Va, USA). Cells were maintained at 37°C under 5% CO_2_, 95% air atmosphere in phenol-red free DMEM/F12 medium with 15% FCS, streptomycin (10 μg/ml) and penicillin (100 U/ml). On the day following plating, BeWo cells were cultured in DMEM/F12 medium with 1% FCS in the presence of various agents such as forskolin (50 μM), adiponectin (25 ng/ml or 250 ng/ml or 500 ng/ml), 17β-estradiol (0.1 μM), progesterone (0.1 μM) and H89 (10 μM).

### Isolation of villous cytotrophoblasts

This study was approved by the local ethical committee (CCP) and informed consent was obtained from each donor before clinical sampling.

First trimester human placental tissues (5-11 week gestational age) were obtained from healthy pregnant women aged between 16 and 36 when undergoing legal abortions. Human placental villous cells were prepared from tips of placental villi as previously described [39]. Placental villi were incubated in HBSS containing 0.125% trypsin, 4.2 mM MgSO4, 25 mM Hepes, and 50 U/ml Dnase type IV at 37°C without agitation. The first 15 min trypsin digestion containing a mix of extravillous and villous CT was discarded. The chorionic villi were then incubated in the same trypsin solution for 15 min at 37°C (three times) and finally washed four times with warm HBSS. Each time, the supernatant containing villous CT was collected after tissue sedimentation, filtered through 100 μm nylon screen, and centrifuged at 200 g for 10 min. Cells were washed twice and then filtered through 40 μm nylon screen. The cell supension was layered over a discontinuous Percoll gradient and centrifuged for 25 min at 1000 g. The layer corresponding to 40-45% percoll containing villous CT was washed twice in DMEM/F12 medium supplemented with 10% FCS. Cells were seeded in 24 well culture plates containing DMEM/F12 medium with 10% FCS, streptomycin (100 μg/ml), penicillin (100 U/ml), and gentamycin (5 μg/ml). Purified villous CT cultures were characterized by positive staining for cytokeratin 7 (CK7) (95% positive cells) and by the observation of cell aggregates and syncytiotrophoblasts from 48 to 72 h. In vitro, purified mononuclear CT spontaneously differentiate to form a multinucleated syncytium after 3 days in culture [39].

### DNA quantification

BeWo cell and villous CT pellets were resuspended in a PBS buffer. DNA content was quantified using an InstaGene Matrix Reagent (Biorad, Hercules, Ca, USA) according to the manufacturer's instructions.

### hCG and cAMP secretions

BeWo cells and villous CT were cultured in DMEM/F12 medium supplemented with 1% FCS with or without adiponectin (250 ng/ml) or forskolin (50 μM) during 24, 48, and 72 h. The culture media were changed every 24 h. The hCG concentrations were measured in the culture medium using an automated immuno-chemiluminescence analyser Architect (Abbott, Rungis, France). In order to compare the secretion of hCG in supernatants, results were normalized to 1 μg of DNA. cAMP concentrations were measured in the culture medium using a cAMP [^3^H] assay system, code TRK432 (GE healthcare, Orsay, France), according to the manufacturer's instructions.

### RT-PCR

BeWo cells or villous CT were seeded in a 12 well culture plate (1.5×10^5 ^cells per well for BeWo cells and 3×10^4 ^cells per well for villous CT) and were cultured in DMEM/F12 medium suplemented with 1% FCS with or without adiponectin (25 or 250 ng/ml) or forskolin (50 μM) or 17β-estradiol (0.1 μM) or progesterone (0.1 μM) for 24 or 48 h. Total RNA (0.1 μg) was extracted and reverse transcribed as previously described [40]. Quantitative PCR was performed using a LightCycler480^® ^instrument from Roche Diagnostics (Basel, Switzerland) using primer sets indicated in table 1. The Second Derivative Maximum Method was used to automatically determine the crossing point (Cp) for individual samples. The two reference genes *TBP *and *β-2-microglobulin *were chosen as previously described [30]. For each sample, the concentration ratio (target/both reference mRNAs) was calculated using the RelQuant Roche software and expressed in arbitrary units. The data were expressed as percentages of control situation. Calibration curves were log-linear over the quantification range with correlation coefficient (r^2^) > 0.99 and efficiency ranging from 1.8 to 2. The intra-assay variability of duplicate crossing point (Cp) values never exceeded 0.2 cycle and the inter-assay variability (CV value) ranged from 1.9 to 4.1% CV values for the 8 or 10 runs of each transcript.

**Table 1:** Primers used for PCR

Primer sets	Sequence	PCR product (bp)
**AdipoR1** Sense Antisense	5' TTC TTC CTC ATG GCT GTG ATG T3' 5'AAG AAG CGC TCA GGA ATT CG 3'	71
**AdipoR2** Sense Antisense	5' ATA GGG CAG ATA GGC TGG TTG A 3' 5' GGA TCC GGG CAG CAT ACA 3'	76
**Syncytin-2** Sense Antisense	5' TCG GAT ACC TTC CCT AGT GC 3' 5' GTA TTC CGG AGC TGA GGT TG 3'	126
**Leptin** Sense Antisense	5' CCA AGA TGG ACC AGA CAC TG 3' 5' GCC ACC ACC TCT GTG GAG TA 3'	220
**TBP** Sense Antisense	5' TGC ACA GGA GCC AAG AGT GAA 3' 5' CAC ATC ACA GCT CCC CAC CA 3'	132
**B-2-microglobulin** Sense Antisense	5' TGC TGT CTC CAT GTT TGA TGT ATC T 3' 5' TCT CTG CTC CCC ACC TCT AAG T 3'	86

### Immunocytochemistry

BeWo cells (4×10^4 ^cells/well), or villous CT (2×10^5 ^cells/well), were plated in a labtech culture device (BD biosciences, San Jose, Ca, USA) and cultured in DMEM/F12 medium supplemented with 1% FCS with or without adiponectin (250 ng/ml) or forskolin (50 μM). After 24, 48 and 72 h treatment, cells were washed three times in PBS buffer and fixed in methanol for 10 min at 4°C. Non-specific IgG binding was blocked by incubation in PBS with 3% BSA for 1 h. Samples were then incubated with primary monoclonal mouse anti-human E-cadherin antibody (Ref: 610181, BD biosciences; San Jose, Ca, USA) (1:200 dilution in PBS BSA 3%) overnight at 4°C. The slides were then rinsed with PBS buffer and incubated with FITC conjugated goat anti-mouse secondary antibody (Ref: SC-2010, Santa Cruz Biotechnology, Inc; Santa Cruz, CA, USA) (1:200 dilution in PBS BSA 3%) for 1 h at room temperature. Cell nuclei were counterstained with DAPI. Syncytium formation was measured by observing the distribution of E-cadherin and nuclei in cells. Control studies were performed using the above described methods using mouse non-specific serum instead of primary antibody.

### RNA interference for AdipoR

Two pairs of small-interfering RNAs (siRNAs) corresponding to different regions of each receptor gene were chemically synthesized by Qiagen (Courtaboeuf, France). The sequences of the sense siRNAs were: for human AdipoR1: AAG GAC AAC GAC TAT CTG CTA and CTG GCT AAA GGA CAA CGA CTA and for human AdipoR2: ACC AAT TTA AGT GAA CAT TTA and CGG CTC TCC TTG AAT AAG AAA. A fluorescently labeled, non-silencing control siRNA was useful for the optimization of transfection conditions and as a control for non-specific silencing effects. For the knockdown experiments, BeWo cells were plated in 24-well dishes at 1×10^5 ^cells/well and cultured for 24 h in medium without antibiotics. Cells were transfected with siRNAs (5 nM/well) using a Lipofectamine RNAiMAX transfection reagent from Invitrogen (Carlsbad, CA, USA) according to the manufacturer's instructions. Adiponectin (500 ng/ml) was added 24 h after transfection. After 72 h of culture, the mRNA expression was analyzed as described above.

### Statistics

Statistical analysis was performed using the raw data from 6 to 10 separate experiments. The non-parametric paired Wilcoxon test was applied to compare one adiponectin concentration effect versus the control situation (without adiponectin) for a given time exposure.

## Results

### Adiponectin effects on biochemical trophoblast differentiation

#### Effects of adiponectin on hCG production in BeWo cells and human villous CT

**Figure 1:**
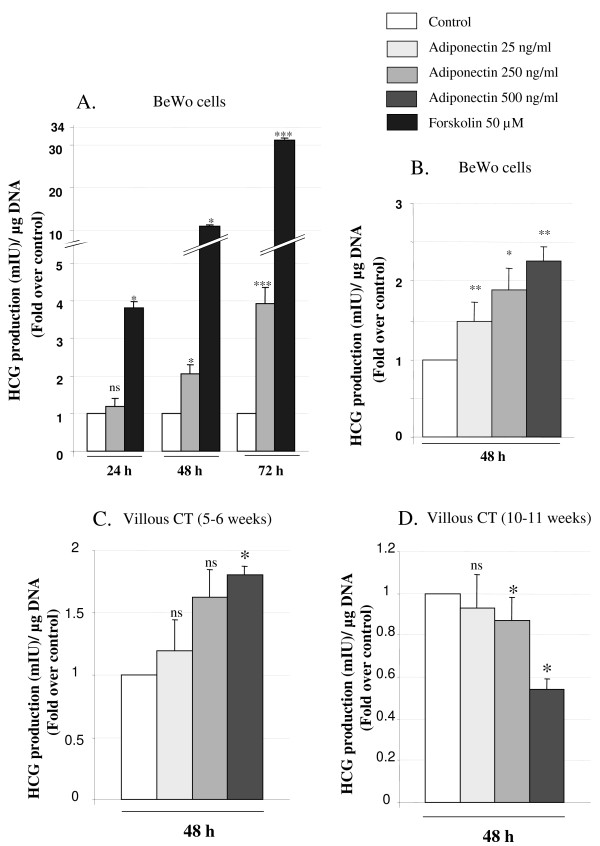
**Regulation of hCG production by adiponectin in BeWo cells and villous CT**. Cells were exposed to adiponectin (25 ng/ml, 250 ng/ml or 500 ng/ml) or forskolin (50 μM) for 24, 48 and 72 h. hCG secretion was measured in the culture medium as described in *Material and Methods *section. The values are the mean ± SEM obtained from 6-8 separate experiments and are expressed as fold-over control value (untreated). For BeWo cells, the control values were: 122 ± 22; 160 ± 52; 119 ± 29 mIU/ml/μgDNA at 24 h, 48 h and 72 h, respectively. **A**. Effect of 24, 48 and 72 h adiponectin (250 ng/ml) exposure on hCG production in BeWo cells. Medium were changed every 24 h. **B**. Effect of 48 h exposure to adiponectin (25, 250 and 500 ng/ml) on hCG production by BeWo cells. **C**. Effect of 48 h exposure to adiponectin (25, 250 and 500 ng/ml) on hCG production by villous cytotrophoblast cells purified from 5-6 week gestational age placentas. **D**. Effect of 48 h exposure to adiponectin (25, 250 and 500 ng/ml) on hCG production by villous cytotrophoblast cells purified from 10-11 week gestational age placentas. For villous CT from 5-6 weeks' gestation, the control values were 2020 ± 822 mIU/ml/μgDNA at 48 h. For villous CT from 10-11 weeks' gestation, the control values were 419 ± 167 mIU/ml/μgDNA at 48 h. *: p < 0.05; ***: p < 0.005; ns : non significant.

Production of hCG by the ST is a marker of biochemical CT differentiation [13]. We measured hCG production in BeWo cells after 24, 48 and 72 h exposure to adiponectin (250 ng/ml). The medium was changed every 24 h. Data presented in Figure 1A show a significant positive effect of adiponectin on hCG production after 48 h exposure (2.05 ± 0.24 fold change), which is even more pronounced after 72 h (3.90 ± 0.42 fold change). A significant positive effect was also observed in the presence of forskolin (50 μM) used as a positive control after 24, 48 and 72 h (Figure 1A). Then, we tested effects of various concentrations of adiponectin (25, 250, 500 ng/ml) after 48 h exposure in BeWo cells. Results in Figure 1B revealed that adiponectin effects on hCG production were already significant at 25 ng/ml (1.47 ± 0.24 fold change) with a maximal effect at 500 ng/ml (2.26 ± 0.22 fold change).

In parallel, we investigated adiponectin effects on CT cells purified from very early first-trimester human placentas (5-6 week gestational age) or early first-trimester human placentas (10-11 week gestational age). In villous CT purified from very early placentas, we observed an increase of hCG production in the presence of adiponectin that was only significant at the maximum concentration (500 ng/ml) (Figure 1C). By contrast, in cells purified from later placentas, adiponectin (250 and 500 ng/ml) reduced hCG production (Figure 1D).

#### Effects of adiponectin on leptin mRNA expression in BeWo cells and human villous CT

It is well established that leptin is produced by ST and can be considered as a marker of biochemical trophoblast differentiation [18]. Accordingly, we investigated the effects of adiponectin on leptin mRNA expression in BeWo cells and CT cells.

**Figure 2:**
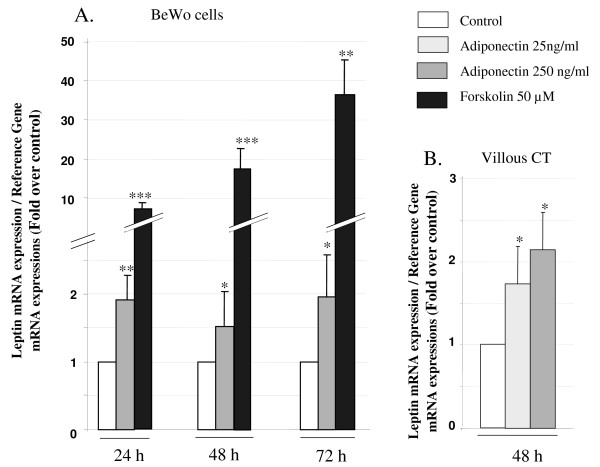
**Regulation of leptin expression by adiponectin in BeWo cells and villous CT**. **A**. BeWo cells were exposed to adiponectin (250 ng/ml) or forskolin (50 μM) for 24, 48 and 72 h. **B**. Villous trophoblast cells were exposed to adiponectin (25 or 250 ng/ml) for 48 h. Total RNA was extracted and analysed by RT-PCR. The values are the mean ± SEM obtained from 6-8 separate experiments and expressed as fold-over control value (untreated). *: p < 0.05; **: p < 0.01; ***: p < 0.005.

As shown in Figure 2A, maximal stimulation of leptin mRNA expression was observed in BeWo cells after 24 h exposure to 250 ng/ml adiponectin (1.91 ± 0.19 fold change). Moreover, this effect was maintained after 48 h (1.53 ± 0.34 fold change) and still persisted up to 72 h exposure (1.27 ± 0.23 fold change). Forskolin (50 μM), which was used as a positive control, induced a very significant up-regulation of leptin gene expression (× 6 after 24 h exposure and up to × 35 after 72 h exposure).

As can be seen in Figure 2B, adiponectin at 25 ng/ml and at 250 ng/ml stimulates leptin mRNA expression in CT cells after 48 h exposure (1.73 ± 0.45 and 2.14 ± 0.45 fold change, respectively).

#### Effects of adiponectin on syncytin-2 mRNA expression in BeWo cells and human villous CT

**Figure 3:**
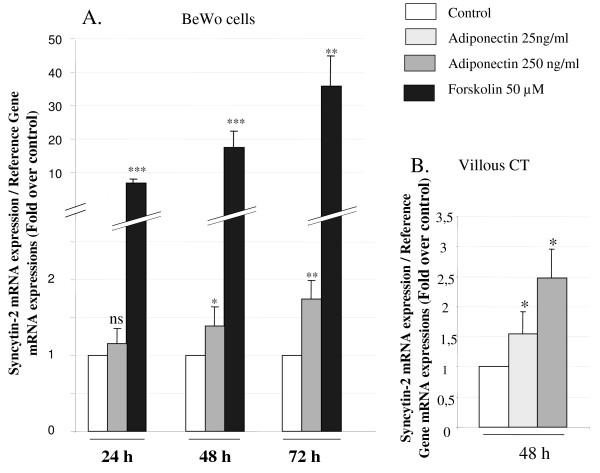
**Regulation of syncytin-2 expression by adiponectin in BeWo cells and villous CT**. **A**. BeWo cells were exposed to adiponectin (250 ng/ml) or forskolin (50 μM) for 24, 48 and 72 h. **B**. Villous trophoblast cells were exposed to adiponectin (25 or 250 ng/ml) for 48 h. Total RNA was extracted and analysed by RT-PCR. The values are the mean ± SEM obtained from 6-8 separate experiments and expressed as fold-over control value (untreated). *: p < 0.05; **: p < 0.01; ***: p < 0.005; ns: non significant.

Syncytin-2, which is up-regulated in ST, has been described as a fusogenic protein involved in trophoblast syncytialization [7,8]. We therefore investigated the influence of adiponectin on syncytin-2 mRNA expression. As shown in Figure 3, exposure to adiponectin (250 ng/ml) significantly increased syncytin-2 mRNA expression in BeWo cells after 24 h exposure (1.16 ± 0.03 fold change). This effect was maintained after 48 and 72 h (1.39 ± 0.23 and 1.75 ± 0.44 fold change, respectively). Forskolin (50 μM), which was used as a positive control, strongly increased syncytin-2 mRNA expression after 24, 48, and 72 h (3.21 ± 0.20, 10.31 ± 0.26 and + 19.06 ± 0.24 fold change, respectively). This stimulatory effect of adiponectin was confirmed using cultured primary trophoblast cells. Adiponectin 250 ng/ml and 500 ng/ml stimulated syncytin-2 mRNA expression in villous CT (+1.55 ± 0.36 and + 2.47 ± 0.56 fold change, respectively).

### Adiponectin effects on morphological trophoblast differentiation

#### Effects of adiponectin on E-cadherin immunostaining in BeWo cells and human villous CT

E-cadherin is a cell adhesion molecule expressed only in isolated CT. During the trophoblast differentiation process, E-cadherin mRNA and protein are down-regulated in association with loss of E-cadherin staining from the surface of fusing cells [4]. Thus, E-cadherin staining is a qualitative marker of trophoblast syncytialization.

**Figure 4:**
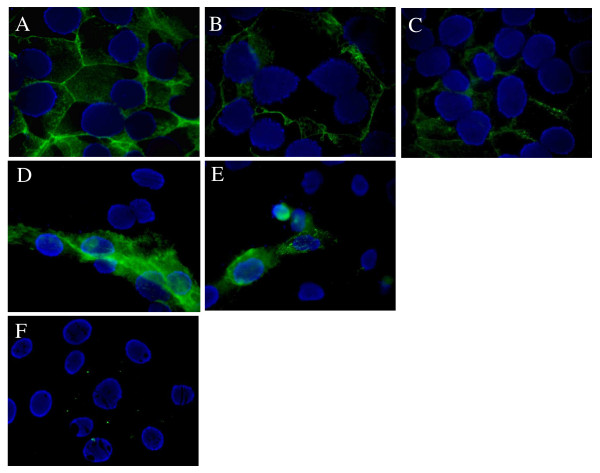
**Regulation of E-cadherin staining by adiponectin in BeWo cells and villous CT**. Cells were exposed to adiponectin (250 ng/ml) or forskolin (50 μM) for 72 h. E-cadherin staining was revealed as described in the *Material and Methods *section. This figure showed one representative among six separate experiments. **(A, B, C) **BeWo cells. **A**. Control (untreated). **B**. Adiponectin. **C**. Forskolin. **(D, E, F) **Villous cytotrophoblasts. **D**. Control (untreated). **E**. Adiponectin. **F**. Negative staining control using mouse non specific serum intead of primary antibody.

To confirm our results on syncytialization, we studied E-cadherin staining in BeWo cells at different time exposures to adiponectin (250 ng/ml) or forskolin (50 μM). The most important effects were observed after 72 h exposure and are presented in Figure 4. Under control conditions (without adiponectin nor forskolin), more than 90% BeWo cells aggregated and showed strong E-cadherin staining at cell boundaries (Figure 4A). Forskolin (50 μM) alone resulted in the disappearance of E-cadherin from cell-cell contact areas (Figure 4C). BeWo cells incubated with adiponectin (250 ng/ml) for 72 h exhibited a loss of E-cadherin staining and a level of syncytialization similar to that in the presence of forskolin (Figure 4B).

E-cadherin staining was also observed in primary villous CT after 48 h exposure to adiponectin. With this time exposure, under control conditions (without adiponectin), we observed that some cells had spontaneously fused and were negative for E-cadherin staining (Figure 4D). Cells exposed to adiponectin for 48 h expressed less E-cadherin than cells cultured in control conditions. Very few cells still expressed E-cadherin (Figure 4E). A negative staining control can be observed on Figure 4F.

### Signalling pathways involved in adiponectin effects on trophoblast differentiation

#### Regulation of AdipoR1 and AdipoR2 mRNA expressions in BeWo cells

**Figure 5:**
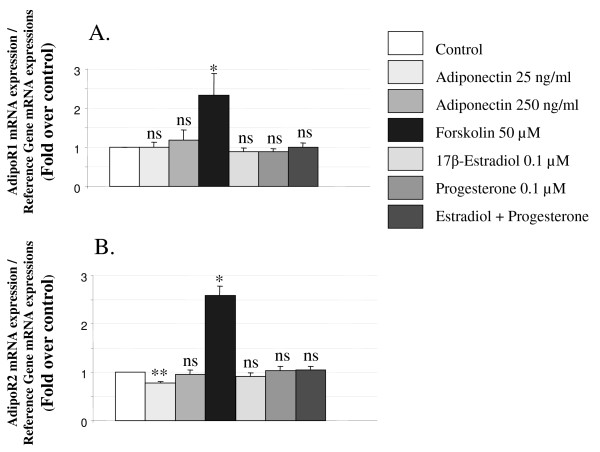
**Regulation of AdipoR mRNA expressions by adiponectin and hormones in BeWo cells**. Cells were exposed to the effectors for 24 h. Total RNA was extracted and analysed by RT-PCR. The values are the mean ± SEM obtained from 6-8 separate experiments and expressed as fold-over control value (untreated). **A**. AdipoR1 mRNA expression. **B**. AdipoR2 mRNA expression. *: p < 0.05; **: p < 0.01; ns : non significant.

To test the impact of AdipoR1 and AdipoR2 on adiponectin effects in BeWo cells, we studied the regulation of AdipoR mRNA expressions by different hormones which are known to play a pivotal role during pregnancy. We demonstrated that forskolin (50 μM) significantly increased the expression of both AdipoR mRNAs after 24 h exposure in BeWo cells (Figure 5A-B). However, as can be seen in Figure 5, 17β-estradiol (0.1 μM) and progesterone (0.1 μM) did not modify AdipoR mRNA expressions in these cells.

Finally, as some studies have described a down-regulation of AdipoR by adiponectin itself [41-43], we studied AdipoR1 and AdipoR2 mRNA expressions in BeWo cells after 24 h exposure to human recombinant adiponectin. We observed a small but significant decrease of AdipoR2 mRNA expression (0.77 ± 0.04 fold change) in the presence of low adiponectin concentration (25 ng/ml) (Figure 5B). However, a higher adiponectin concentration (250 ng/ml) did not modify AdipoR1 and AdipoR2 mRNA expressions in BeWo cells (Figure 5A-B).

#### SiRNA down-regulation of AdipoR in BeWo cells

**Figure 6:**
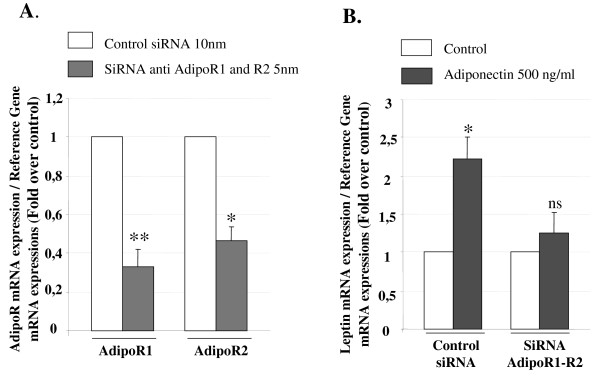
**Role of specific adiponectin receptors in differentiating effects of adiponectin in BeWo cells**. Effects of adiponectin in AdipoR-deficient BeWo cells. Cells were cultured for three days in the presence of 5 nM AdipoR1/R2 or 10 nM control siRNA. **A**. Level of AdipoR in transfected cells. Total RNA was extracted and analyzed by RT-PCR as described in *Material and Methods *section. Results are the means ± SEM of 8 experiments and are expressed as a percentage of the control (non-silencing). **B**. Effect of adiponectin on the induction of leptin expression. AdipoR-depleted cells with 5 nM of siRNA were or were not exposed to adiponectin (500 ng/ml) for 48 h. Results are expressed as fold-over control value (without adiponectin). Each bar represents the mean ± SEM of six separate experiments. *: p < 0.05; **: p < 0.01; ns : non significant.

In these experiments, we used two different siRNA for each receptor and we observed a decrease of 67 and 54% in AdipoR1 and R2 expression, respectively, after 72 h of transfection (Figure 6A). Furthermore, as shown in Figure 6B, this partial suppression of AdipoR1 and R2 with siRNA inhibited the increasing leptin mRNA expression by adiponectin.

#### Transduction pathways involved in adiponectin effects on BeWo cells

The classical signal transduction pathway involved in CT differentiation is the activation of the adenylate cyclase -cAMP- PKA pathway [44]. We tested the impact of the PKA pathway on the induction of hCG secretion by adiponectin in BeWo cells using a PKA transduction pathway inhibitor.

**Figure 7:**
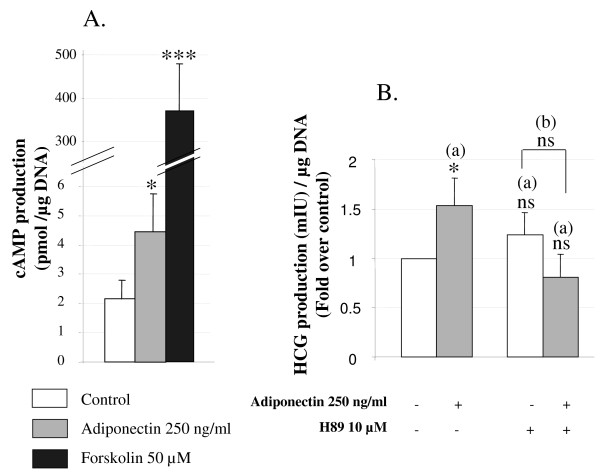
**Role of PKA pathway in differentiating effects of adiponectin in BeWo cells**. BeWo cells were exposed to adiponectin (250 ng/ml) or PKA inhibitor H89 (10 μM) or adiponectin with H89 or foskolin alone (50 μM) for 48 h. **A**. Effects of adiponectin on cAMP production by BeWo cells was measured in the culture medium as described in the *Material and Methods *section. The values are the mean ± SEM obtained from 9-10 separate experiments. **B**. Effect of H89 on adiponectin hCG production by BeWo cells. hCG secretion was measured in the culture medium as described in *Material and Methods *section. The values are the mean ± SEM obtained from 7-8 separate experiments and expressed as fold-over the control value (untreated). *: p < 0.05; ***: p < 0.001; ns : non significant. (a): vs control (without H89 nor adiponectin). (b): adiponectin + H89 vs H89 alone.

We demonstrated that treatment of BeWo cells with adiponectin (250 ng/ml) for 48 h increased cAMP production as compared to the control situation (2.05 ± 0.29 fold change; Figure 7A). Forkolin, used as a positive control, strongly increased cAMP levels. Moreover, as shown in Figure 7B, treatment of BeWo cells with adiponectin for 48 h in the presence of the specific inhibitor H89 (10 μM) for PKA pathway suppressed the positive effect of adiponectin on hCG secretion. In our experimental conditions, addition of H89 alone induced a slight increase of hCG production in these cells (1.39 ± 0.21 fold change).

## Discussion

Many factors involved in the regulation of trophoblast differentiation have been described. Some of them are also involved in trophoblast proliferation. We have recently shown that adiponectin exerts anti-proliferative effects on trophoblast cells [30]. In the present study, we provide new evidence that adiponectin also promotes a trophoblast differentiation process.

We have examined direct adiponectin effects on BeWo cell and villous CT differentiation by studying biochemical and morphological markers of the ST. We have found that adiponectin was able to i) up-regulate expression of fusion markers as syncytin-2 and ii) reduce E-cadherin membrane staining, indicating that adiponectin promotes the syncytialization of trophoblast cells. Moreover, we have shown for the first time that adiponectin induces a strong increase of leptin expression in BeWo cells and human villous CT. In this way, adiponectin could potentiate the positive effects of leptin on proliferation and invasion of trophoblast cells [21,22,45].

In BeWo cells, adiponectin also mediates a modest but reproducible induction of hCG production. To investigate the physiological relevance of this result, we examined the effects of adiponectin on hCG production in villous CT. We observed that adiponectin exerts a gestational age-dependent dual action on first trimester placentas. Like in BeWo cells, we observed an up-regulation of hCG production by adiponectin in villous CT purified from very early placentas (5-6 week gestational age). By contrast, in cells purified from later placentas (10-11 week gestational age), adiponectin decreased hCG production. This last result is in accordance with another study showing a down-regulation of hCG production by adiponectin on the ST of term placentas [46]. Moreover, similar results were observed with the placental growth factor EGF, that has gestationnal age-dependent effects in first trimester placentas [47]. This switch could be related to the oxygen exposure during placentation. Indeed, it is well established that during early pregnancy, placentation occurs in a relative hypoxic environment. After 10-12 weeks' gestation, the intervillous space opens to maternal blood and results in exposure of the trophoblasts to increased oxygen levels [2]. In this context, we can hypothesize that the dual effect of adiponectin on hCG production between 5-6 weeks and 10-11 weeks of gestation could be dependent on signalling pathways and/or transcriptional factors sensitive to oxygen concentrations [48]. Experiments are currently in progress in our laboratory to test this hypothesis.

We have thus shown that adiponectin is a new regulator of leptin and hCG production, which are both essential placental hormones. Moreover, it was recently shown that leptin expression is up-regulated by hCG in the trophoblastic cell line BeWo and in placental explants [49] and, inversely, that leptin increases hCG production in trophoblast and adipose cells [50-52]. Adiponectin, through direct or indirect actions, could strengthen this amplification loop between hCG and leptin. Moreover, the positive effect of adiponectin on hCG expression might be reinforced by the up-regulation of AdipoR1 by hCG itself (data not shown) [53]. This last finding is in accordance with an *in vivo *study showing an up-regulation of AdipoR1 -specifically by hCG- in rat ovaries [53]. Silencing the AdipoR1 and AdipoR2 genes suppressed adiponectin effects on leptin expression. This result suggests a critical impact of AdipoR in adiponectin regulation of trophoblast differentiation.

Biological effects of adiponectin are initiated by AdipoR1 and AdipoR2 inducing the activation of protein kinases, mainly the AMPK but also the PKA pathways [32]. The rise of intracellular cAMP production by the PKA pathway is widely described as the main signal mediating trophoblast differentiation [44]. Thus, we used a specific inhibitor (H89) to investigate the impact of PKA signalling pathway in adiponectin effects in BeWo cells. We show that the addition of H89 suppressed the positive effect of adiponectin on hCG secretion. Moreover, adiponectin increases cAMP release in BeWo cells, reinforcing PKA signalling impact. However, this increase was more pronounced in the presence of forskolin and could explain the difference between adiponectin and forskolin effects on hCG production.

Finally, we found that AdipoR1 and AdipoR2 mRNA expressions were increased by forskolin, suggesting a cAMP dependent mechanism. This up-regulation of the AdipoR could maintain a higher sensitivity to adiponectin in trophoblast cells and thus promote the positive effects of adiponectin on trophoblast differentiation.

A recent study showing that adiponectin induces differentiation and fusion of muscle cells via AMPK pathway [54]. Our results show that adiponectin can promote cell fusion in another cell type.

The process of syncytialization is linked to the early stages of the apoptotic cascade within cytotrophoblast cells [55]. In particular, initiator caspase 8 and caspase 14 are involved in this process [56,57]. Adiponectin has been described as a pro-apoptotic factor in many cell types, including breast cancer cells [58] and endometrial cells [17]. Further experiments will be needed to establish whether adiponectin effects on differentiation are also mediated by these caspases.

Inappropriate trophoblast differentiation is a potential cause of the aetiology of pre-eclampsia and fetal growth restriction. Altered plasma adiponectin concentrations have been described in women with pre-eclampsia [59], reinforcing its possible impact on the control of trophoblast differentiation.

In conclusion, our study reveals a novel function for adiponectin in modulating trophoblast differentiation.

## Abbreviations

AMPK: AMP Activated Protein Kinase; PKA: Protein Kinase A; MAPK: Mitogen Activated Protein Kinase; cAMP: cyclic Adenosine Mono Phosphate; RT-PCR: Reverse Transcription-Polymerase Chain Reaction; CK7: cytokeratine 7.

## Competing interests

The authors declare that they have no competing interests.

## Authors' contributions

MND, EDS, RP and PdM conceived, designed and coordinated much of this project. The experiments were carried out by DB, MND, EDS and MCL. Data analysis was performed by DB, MND, EDS and RP. DB wrote the manuscript with participation from MND and EDS. MND, EDS, RP and PdM reviewed the manuscript. All authors read and aproved the final manuscript.
